# MNSFβ regulates placental development by conjugating IGF2BP2 to enhance trophoblast cell invasiveness

**DOI:** 10.1111/cpr.13145

**Published:** 2021-10-20

**Authors:** Qian Yang, Yeling Ma, Yanlei Liu, Xuan Shao, Wentong Jia, Xin Yu, Yu‐xia Li, Long Yang, Wenwen Gu, Haibin Wang, Jian Wang, Yan‐Ling Wang

**Affiliations:** ^1^ NHC Key Lab of Reproduction Regulation (Shanghai Institute for Biomedical and Pharmaceutical Technologies) Fudan University Shanghai China; ^2^ State Key Laboratory of Stem Cell and Reproductive Biology Institute of Zoology Institute for Stem Cell and Regeneration Chinese Academy of Sciences Beijing China; ^3^ Medical College Shaoxing University Shaoxing China; ^4^ Center for Reproductive Medicine School of Medicine Cheeloo College of Medicine Shandong University Jinan China; ^5^ Beijing Institute for Stem Cell and Regenerative Medicine Beijing China; ^6^ University of Chinese Academy of Sciences Beijing China; ^7^ Fujian Provincial Key Laboratory of Reproductive Health Research School of Medicine Xiamen University Xiamen China

**Keywords:** IGF2BP2, MNSFβ, placenta, preeclampsia, trophoblast cell invasion

## Abstract

**Objectives:**

Success in pregnancy in mammals predominantly depends on a well‐developed placenta. The differentiation of invasive trophoblasts is a fundamental process of placentation, the abnormalities of which are tightly associated with pregnancy disorders including preeclampsia (PE). Monoclonal nonspecific suppressor factor beta (MNSFβ) is an immunosuppressive factor. Its conventional knockout in mice induced embryonic lethality, whereas the underlying mechanism of MNSFβ in regulating placentation and pregnancy maintenance remains to be elucidated.

**Methods:**

Trophoblast‐specific knockout of *MNSFβ* was generated using Cyp19‐Cre mice. *In situ* hybridization (ISH), haematoxylin and eosin (HE), immunohistochemistry (IHC) and immunofluorescence (IF) were performed to examine the distribution of MNSFβ and insulin‐like growth factor 2 mRNA‐binding protein 2 (IGF2BP2) at the foeto‐maternal interface. The interaction and expression of MNSFβ, IGF2BP2 and invasion‐related molecules were detected by immunoprecipitation (IP), immunoblotting and quantitative real‐time polymerase chain reaction (qRT‐PCR). The cell invasion ability was measured by the Transwell insert assay.

**Results:**

We found that deficiency of MNSFβ in trophoblasts led to embryonic growth retardation by mid‐gestation and subsequent foetal loss, primarily shown as apparently limited trophoblast invasion. In vitro experiments in human trophoblasts demonstrated that the conjugation of MNSFβ with IGF2BP2 and thus the stabilization of IGF2BP2 essentially mediated the invasion‐promoting effect of MNSFβ. In the placentas from MNSFβ‐deficient mice and severe preeclamptic (PE) patients, downregulation of MNSFβ was evidently associated with the repressed IGF2BP2 expression.

**Conclusions:**

The findings reveal the crucial role of MNSFβ in governing the trophoblast invasion and therefore foetal development, and add novel hints to reveal the placental pathology of PE.

## Graphical abstract

In placental mammals, the appropriate differentiation of placental trophoblast cell along the invasive pathway predominantly governs placentation and reproductive success. Here, we reveal the crucial role of MNSFβ in governing trophoblast cell invasion via interaction with IGF2BP2. Attenuated expression of placental MNSFβ and IGF2BP2 leads to limited trophoblastic invasiveness and pregnancy disorder including preeclampsia (PE).

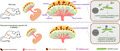

## 1 INTRODUCTION

As a transient organ that is particularly formed during pregnancy in mammals, the placenta acts as an indispensable safeguard for both foetal growth and maternal health.^1^, ^2^ The recent integrative investigation in genetically manipulated mice has strongly revealed that defects in placentation are highly prevalent in abnormal embryo development.^3^ Placental trophoblast cell differentiation along the invasive pathway is one of the fundamental processes for placental development. The invasive extravillous trophoblasts (EVTs) infiltrate into decidua to anchor the embryo to the uterine wall, reconstruct uterine blood vessels to ensure blood supply and establish an immune‐tolerant environment through interaction with maternal immune cells. Defects in trophoblast cell invasiveness and subsequent insufficient remodelling of maternal spiral arteries have been well recognized as the dominant pathological factors of pregnancy‐associated disorders including PE.^2^, ^4^, ^5^

We previously identified a remarkable differential gene, *MNSFβ*, in mouse implantation site,^6^, ^7^ which encodes a non‐antigen‐specific immunosuppressive factor with 97.8% homology between human and mouse. Structurally, MNSFβ consists of 133 amino acids which form a ubiquitin‐like (Ubi‐like) domain and a ribosomal S30 domain.^8^, ^9^, ^10^ Conventional knockout of *MNSFβ* gene in mice led to embryonic lethality of homozygotes (MNSFβ^−/−^) and significantly retarded *in utero* growth of heterozygotes (MNSFβ^+/−^) after embryonic day 10.5 (E10.5).^11^ In addition, we found the invasion‐promoting effect of MNSFβ on human trophoblasts, and its markedly decreased expression in the placentas of recurrent miscarriage patients.^11^, ^12^ These evidences highly indicate the dominant roles of MNSFβ in regulating trophoblast differentiation and therefore governing pregnancy outcomes.

To further address the regulatory mechanisms of MNSFβ in the process of placental development, here we generated a mouse model with specific deletion of *MNSFβ* gene in placental trophoblasts (PHTs) using the *Cyp19*‐*Cre* tool. Phenotypical analysis of embryonic and placental growth as well as trophoblast differentiation was conducted. Glutathione S‐transferase (GST) pull‐down, mass spectrometry and invasion assay in human trophoblast cells were carried out to determine the interaction between MNSFβ and IGF2BP2 that can regulate trophoblast cell invasiveness. What's more, the aberrant expressions of MNSFβ and IGF2BP2 in severe PE placentas were examined. The findings reveal MNSFβ as a novel factor governing trophoblast cell invasiveness and therefore foetal development.

## 2 MATERIALS AND METHODS

### 2.1 Generation of placenta‐specific knockout of MNSFβ in mice

*MNSFβ^loxp^* ^/^ *^loxp^* mice were generated previously,^11^ and *Cyp19*‐*Cre* transgenic mice were kind gift from Professor Gustavo Leone′s group.^13^ According to the previous report,^13^ the CYP19 promoter can lead to Cre expression in all derivatives of trophoblast stem cells from E6.5 on, and only less than 4.5% foetuses exhibit weak‐to‐moderate Cre expression in areas of foetal skin, lens of the eye and corpus callosum of the hindbrain. The mice were housed in the Animal Care Facility in the Institute of Zoology (IOZ), Chinese Academy of Sciences (CAS), and the experimental procedure was approved by the Animal Welfare and Ethics Committees in IOZ. All mice were housed under a 12 h light/12 h dark cycle (light from 07:00 AM to 19:00 PM, temperature [23–25°C] and relative humidity [40%–60%]), with adequate water and food supply. For the mating experiment, 8–10‐week‐old male and virgin female mice were cohoused from 5:00 PM to 8:00 AM, and the morning when vaginal plug was detected was recorded as embryonic day (E) 0.5 of pregnancy. To get placental‐specific knockout of MNSFβ in mice, *Cyp19*‐*Cre* ^+/−^/ *MNSFβ^loxp^* ^/+^ mice (F1) were generated by crossing *MNSFβ^loxp^* ^/^ *^loxp^* mice with *Cyp19*‐*Cre* transgenic mice (F0), and the F1 mice were crossed with *MNSFβ^loxp^* ^/^ *^loxp^* mice to get F2 foetuses including Control (*MNSFβ^loxp^* ^/+^ or *MNSFβ^loxp^* ^/^ *^loxp^*), Het (*Cyp19*‐*Cre* ^+/−^/ *MNSFβ^loxp^* ^/+^) and cKO (*Cyp19*‐*Cre* ^+/−^/ *MNSFβ^loxp^* ^/^ *^loxp^*). After sacrificed by cervical dislocation at E10.5 or E13.5, the foetuses and placentas were weighed separately, and the placental tissues were fixed in 4% paraformaldehyde (PFA) (Sigma‐Aldrich) and subjected to paraffin or frozen embedding. Tail tips of the foetuses were collected for genotyping by PCR with specific primers for Cre (Forward primer, 5′‐CCACGACCAAGTGACAGCAA‐3′; Reverse primer, 5′‐TGACCAGAGTCATCCTTAGCG‐3′) and MNSFβ (Forward primer, 5′‐CACTTCTTCCTCTTTCTTGACTCC‐3′; Reverse primer, 5′‐ CGATCTAAAGTCCCTAGAAGGCAC‐3′).

### 2.2 Collection of human placental and decidual tissues

The study was approved by the Local Ethical Committees in IOZ, the Second Hospital Affiliated to Tianjin Medical University (Tianjin, China) and Peking University Third Hospital (Beijing, China). Written informed consent was obtained from the enrolled pregnant women. The decidual and villous tissues from healthy pregnant women at early gestation were collected at the Second Hospital Affiliated to Tianjin Medical University (Tianjin, China), and the clinical features of these pregnant women are summarized briefly in Table S1. Human placental tissues from patients with severe PE (PE, *n* = 7) or unexplained preterm labour (PTL, *n* = 7) were collected at Peking University Third Hospital (Beijing, China). The placentas from PTL were used as the gestation‐matched controls for severe PE, as described previously.^14^ The clinical features of the pregnant women enrolled in this study are summarized in Table S2. Severe PE was defined as a pregnancy having no history of preexisting or chronic hypertension but showing systolic blood pressure of ≥160 mmHg or diastolic blood pressure of ≥110 mmHg on at least two occasions, accompanied by significant proteinuria (≥2 g/24 h or 3+ by dipstick in two random samples collected at >4 h intervals) or problems in multiple organs (such as pulmonary oedema, seizures, oliguria, abnormal liver enzymes associated with persistent epigastric or right upper quadrant pain, or persistent and severe central nervous system (CNS) symptoms) after the 20th week of gestation.^15^ PTL was defined as labour earlier than the 34th week, but without clinical or pathological features of other maternal or placental complications. Specimens of the placenta were obtained immediately after caesarean section and subjected to snap freezing.

### 2.3 Cell culture and treatment

The human choriocarcinoma cell line JEG3 was purchased from American Type Culture Collection (ATCC). The HTR8/SVneo cell line was kindly provided by Dr. C.H. Graham at Queen’s University, Canada. The JEG3 cells were maintained in Dulbecco's Modified Eagle's Medium (DMEM) (Gibco,) supplemented with 10% foetal bovine serum (FBS, Gibco), and the HTR8/SVneo cells were cultured in Roswell Park Memorial Institute (RPMI) 1640 medium (Gibco) supplemented with 10% FBS (Gibco).

Human trophoblast stem cells (hTSCs) were derived from first trimester placental villi according to a previous publication.^16^ hTSCs were cultured in a 6‐well plate pre‐coated with 5 μg/ml of collagen type IV (Col IV) (Sigma‐Aldrich) with 2 ml of tryptose sulphite cycloserine (TSC) medium: DMEM/F12 (nutrient mixture) (Gibco) supplemented with 0.1mM 2‐mercaptoethanol (Sigma‐Aldrich), 0.2% FBS (Gibco), 0.5% Penicillin‐Streptomycin (Gibco), 0.3% bovine serum albumin (BSA) (Wako), 1% insulin‐transferrin‐selenium ethanolamine (ITS‐X) supplement (Wako), 1.5 μg/ml of L‐ascorbic acid (Wako), 50 ng/ml of epidermal growth factor (EGF) (Wako), 2 μM CHIR99021 (Wako), 0.5 μM A83‐01 (Wako), 1 μM SB431542 (Wako), 0.8 mM valproic acid (VPA) (Wako) and 5 μM Y27632 (Wako).

Transient transfection experiments were carried out using Lipofectamine 2000 according to the manufacturer's instruction (Invitrogen). The small interfering RNA (siRNA) duplexes were generated by GenePharma. Sequences for siRNA were as follows: MNSFβ (sense, 5′‐CCAAACAGGAGAAGAAGAATT‐3′; antisense, 5′‐UUCUUCUUCUCCUGUUUGGTT‐3′); IGF2BP2 (sense, 5′‐GCGAAAGGAUGGUCAUCAUTT‐3′; antisense, 5′‐AUGAUGACCAUCCUUUCGCTT‐3′); negative control (NC) (scrambled sequence) (sense, 5′‐UUCUCCGAACGUGUCACGUTT‐3′; antisense, 5′‐ACGUGACACGUUCGGAGAATT‐3′). Following 24 h or 48 h of transfection, total RNAs or proteins were extracted for qRT‐PCR or immunoblotting.

### 2.4 Haematoxylin and eosin (HE) staining and immunohistochemistry (IHC) or immunofluorescence (IF)

Freshly collected tissues were subjected to embedding in an optimal cutting temperature (OCT) compound (Sakura Finetek) or fixation in 4% paraformaldehyde (PFA) followed by routine dehydration and paraffin embedding. Frozen sections at 8 µm or paraffin sections at 5 μm were subjected to staining with Mayer's haematoxylin and eosin Y solutions (Sigma‐Aldrich), or antigen retrieval followed by incubation with specific antibody against cytokeratin 7 (CK7, 1:300, ab75813, Abcam), IGF2BP2 (1:200, 11601–1‐AP, Proteintech) or MNSFβ (1:500, Beijing ComWin Biotech Co. Ltd.). Negative controls (NCs) were incubated with immunoglobulin G (IgG), instead of primary antibody. For IHC, the sections were further incubated with horseradish peroxidase (HRP)‐conjugated secondary antibodies (PV‐6001, Zhongshan) and visualized with 3,3'‐diaminobenzidine (DAB) (ZLI‐9019, Zhongshan). The staining results were recorded on a light microscope (DP72, Olympus) and analysed using Image‐Pro Plus Version 6 software. For IF, the sections were incubated with fluorescein isothiocyanate (FITC)‐conjugated or tetramethylrhodamine (TRITC)‐conjugated secondary antibody (1:200, ZF‐0311, ZF‐0313, Zhongshan), with cell nuclei being stained with 4,6‐diamidino‐2‐phenylindole (DAPI; 28718–90–3, Sigma‐Aldrich). The results were recorded on a Zeiss LSM780 confocal microscope system (Zeiss, Germany) and processed with ZEN 2012 software (Zeiss).

### 2.5 In situ hybridization (ISH)

MNSFβ‐specific riboprobe was designed to recognize nucleotides 1–399 of *Mus musculus* MNSFβ mRNA (messenger RNA; GenBank Accession No. BC058691.1). The sense probe and antisense probe were in vitro transcribed with mouse placenta complementary DNA (cDNA) as template using forward primer: 5′‐GCGTCCGCGGGCTAGTA‐3′ and reverse primer: 5′‐TATTAGCATGGCAGGGTGGC‐3′. The digoxin‐labelled riboprobe was synthesized according to the manufacturer's instruction (Roche). The ISH was carried out, as described previously.^17^, ^18^ Briefly, serial frozen sections at 10 μm were fixed in 4% PFA, treated with protease K and hybridized with sense or antisense probes for MNSFβ at 55°C. The sections were washed in saline‐sodium citrate buffer, followed by incubation with anti‐digoxin antibody (1:200, 11093274910, Roche). 5‐Bromo‐4‐chloro‐3‐indolyl‐phosphate/nitro blue tetrazolium (BCIP/NBT) (Promega) was used as substrate to visualize the signals. The staining results were recorded on a light microscope (DP72, Olympus) and analysed using Image‐Pro Plus Version 6 software.

### 2.6 Quantitative real‐time PCR (qRT‐PCR)

Total RNA was extracted from tissues or cells using TRIzol reagent (Invitrogen), and 2 µg of total RNA was subjected to cDNA synthesis. Real‐time PCR was carried out using LightCycler480 sequence detection system (Roche). The primers were listed as follows: Homo *MNSFβ*: Forward primers: 5′‐ACTCCATCTTCGCGGTAGC‐3′; Reverse primers: 5′‐GGAGCACGACTTGATCTTCC‐3′. Homo *Igf2bp2*: Forward primers: 5′‐TCTTTGGGGACAGGAAGCTG‐3′; Reverse primers: 5′‐GTAGTCCACGAAGGCGTAGC‐3′. Homo *Gapdh*: Forward primers: 5′‐TGAAGGTCGGAGTCAACGGA‐3′; Reverse primers: 5′‐CCTGGAAGATGGTGATGGGAT‐3′. Mus *Prl3a1*: Forward primers: 5′‐GAAGGAGCCTGCAAGACCAT‐3′; Reverse primers: 5′‐CATCTGCCAGTCCCATCCAA‐3′. Mus *Prl3b1*: Forward primers: 5′‐CCAGAAAACAGCGAGCAAGT‐3′; Reverse primers: 5′‐AGGTACATGTGGAAGAGCAGC‐3′. Mus *Prl2c2*: Forward primers: 5′‐TGAGGAATGGTCGTTGCTTT‐3′; Reverse primers: 5′‐TCTCATGGGGCTTTTGTCTC‐3′. Mus *Prl3d1*: Forward primers: 5′‐TGGTGTCAAGCCTACTCCTTT‐3′; Reverse primers: 5′‐CAGGGGAAGTGTTCTGTCTGT‐3′. Mus *Gapdh*: Forward primers: 5′‐GGAGAAACCTGCCAAGTATGATG‐3′; Reverse primers: 5′‐AAGAGTGGGAGTTGCTGTTGAAG‐3′. The relative level of the detected gene was normalized to endogenous *Gapdh*. The mRNA expression was calculated using the 2^−ΔΔCT^ method, where ΔCT indicates the subtraction of the threshold cycle (CT) for Gapdh from that for the interested gene.^19^

### 2.7 Plasmid construction

The coding region for IGF2BP2 (GenBank Accession No. BC021290.2) was amplified from human placental cDNA and cloned into pcDNA4 vector (Promega) at KpnI and EcoRI sites. The construct was named pcDNA4‐IGF2BP2. The amplified primers were as follows: Forward primers: 5′‐ATGAACAAGCTTTACATCG‐3′; Reverse primers: 5′‐TGGAAGGGCTACATTCAT‐3′. The construct sequence was confirmed by DNA sequencing.

### 2.8 Transwell insert assay

The cell invasion assay was conducted, as previously described.^20^ Briefly, 24‐well fitted Transwell inserts (3422, Costar) were pre‐coated with growth factor‐reduced Matrigel matrix (356230, BD Biosciences) at a concentration of 200 µg/ml. Following 24 h of siRNA or plasmid transfection, JEG3 or HTR8/SVneo cells were harvested and seeded at 5 x 10^4^/100 µl cells in each insert containing FBS‐reduced medium. After 32 h of incubation, the cells were fixed in 4% PFA and stained with crystal violet. The non‐invaded cells on the upper surface of membrane were removed using a cotton swab, and the cells on the lower surface were counted. In parallel to Transwell insert assay, Cell Counting Kit‐8 (CCK‐8) (Dojindo) assay in a 96‐well plate was performed to measure cell viability for the same batch of cells according to the manufacturer's instructions (Figure S3). The number of invaded cells was adjusted by the total cell number to measure the invasion index.

### 2.9 Immunoprecipitation (IP) and immunoblotting analysis

Total protein was extracted from frozen tissues or cultured cells using radioimmunoprecipitation (RIPA) lysis buffer (Beyotime) with protease inhibitor cocktails (Sigma‐Aldrich), and 30 µg of total protein was separated by 12% sodium dodecyl sulphate‐polyacrylamide gel electrophoresis (SDS‐PAGE) and transferred to polyvinylidene fluoride (PVDF) membranes (Amersham Pharmacia Biotech). After blocking in 5% BSA, the membranes were incubated with primary antibodies: against MNSFβ (1:2000, Beijing ComWin Biotech Co. Ltd.); IGF2BP2 (1:2000, 11601–1‐AP, Proteintech); and β‐actin (1:5000, KM9001T, Tianjin Sungene Biotech). Following a wash with 0.1% phosphate‐buffered saline/Tween (PBST), the membranes were incubated with HRP‐conjugated secondary antibody (0.1 µg/ml; Promega). The signals were visualized with the enhanced chemiluminescence Western blot analysis system (Pierce). The relative densities of the detected proteins were normalized by the density value of β‐actin in the same blot.

For IP, total proteins (1–2 mg) were extracted using the IP lysis buffer (Roche). After centrifugation at 10625 *g*, the lysates were incubated with antibody against MNSFβ, IGF2BP2 or normal IgG (BD0051, Bioworld) followed by incubation with Protein A/G magnetic beads (Santa Cruz). The beads were washed with IP washing buffer and eluted with 1x SDS loading buffer. The samples were subjected to SDS‐PAGE and immunoblotting for antibody against IGF2BP2 (1:1000, 11601–1‐AP, Proteintech), MNSFβ (1:1000, Beijing ComWin Biotech Co. Ltd) or ubiquitin (Ub, 1:200, sc‐8017, Santa Cruz).

### 2.10 Statistical analysis

The statistical analysis was performed with GraphPad Prism version 5.01 (GraphPad Software). Data were shown as mean ± SEM according to at least three independently repeated experiments. The differences between groups were analysed by independent Student's *t*‐test or unpaired one‐way analysis of variance (ANOVA) with Sidak's correction. The P values less than 0.05 were considered statistically significant.

## 3 RESULTS

### 3.1 Expression pattern of MNSFβ at the foeto‐maternal interface of human and mouse

**FIGURE 1:**
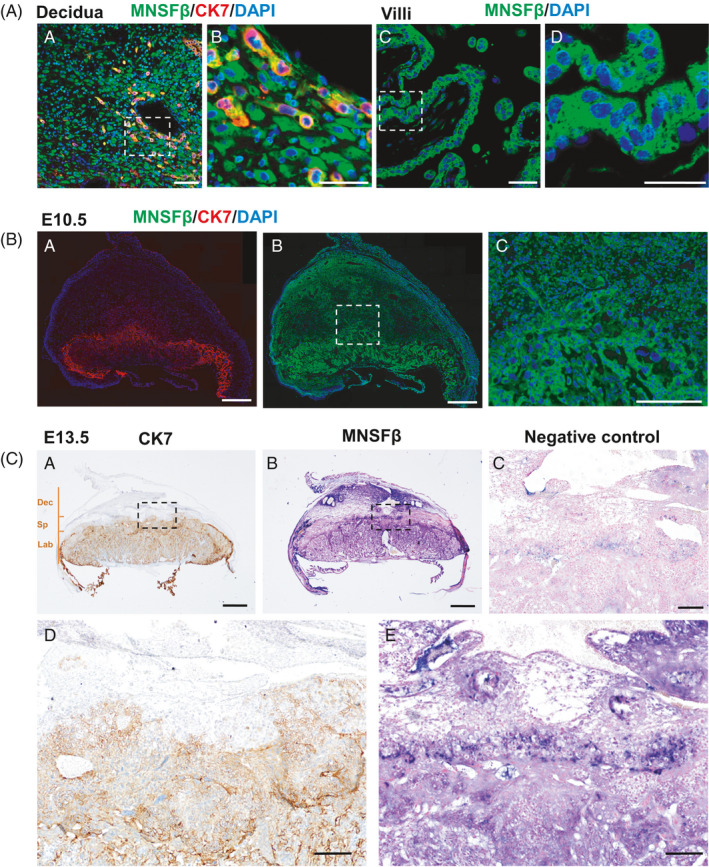
Localization of monoclonal nonspecific suppressor factor beta (MNSFβ) at the foeto‐maternal interface in human and mouse. (A: a and c), Immunofluorescence (IF) staining of MNSFβ (green) and/or cytokeratin 7 (CK7) (red) in human decidua and placental villi at weeks 7–9 of normal gestation. (b and d), Enlargement of the areas, as indicated in panels a and c, separately. (B: a‐b), IF staining of MNSFβ (green) and/or CK7 (red) in mouse placentas at embryonic day 10.5 (E10.5). (c), Enlargement of the area, as indicated in panel b. (C: a‐b), Immunohistochemistry (IHC) of CK7 (brown) and in situ hybridization (ISH) for MNSFβ (antisense probe, blue) in mouse placentas at E13.5. (d and e), Enlargement of the areas, as indicated in panels (a and b), separately. Hybridization with sense probe was performed as negative control and is shown in (c). Three functional layers at the foeto‐maternal interface – decidua (Dec), sponge zone (Sp) and labyrinth (Lab) were lined out in (a). Scale bars indicate 200 μm in (A) and (c) of (B), 600 μm in (a‐b) of (B), 500 μm in (a‐b) of (C) or 100 μm in (c‐e) of (C)

Immunofluorescent staining and/or ISH for MNSFβ was performed to clearly demonstrate its distribution at the foeto‐maternal interface of human and mouse. In human placental villous and decidual tissues at early gestation, immunofluorescent signals were intensively observed in villous cytotrophoblasts (CTBs), syncytiotrophoblasts (STBs) and EVTs, as well as many other cell types in placenta and decidua (Figure 1A). At the mouse foeto‐maternal interface at E10.5 and E13.5, the MNSFβ expression could be extensively observed in multiple trophoblast subsets and other cell types within the three functional layers – decidua (Dec), sponge zone (Sp) and labyrinth (Lab) (Figure 1B,C). The data are in consistence with our recent report showing the wide distribution of MNSFβ in human placenta,^12^ indicating the participation of this protein in multiple cell events at the foeto‐maternal interface.

### 3.2 Placental‐specific deficiency in MNSFβ leads to retarded embryonic and placental development at early‐to‐mid gestation in mice

**FIGURE 2:**
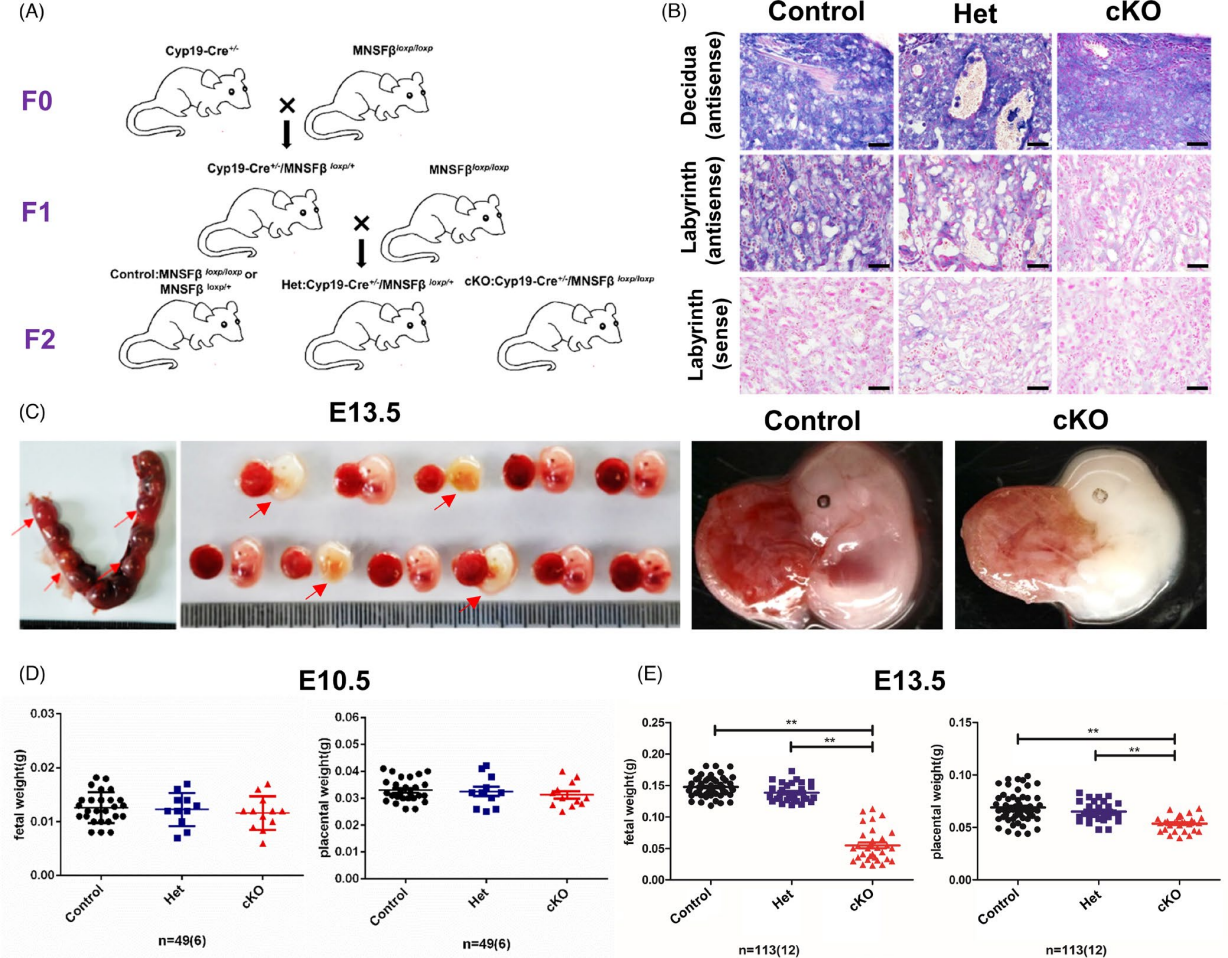
Deletion of monoclonal nonspecific suppressor factor beta (MNSFβ) in placental trophoblast cells results in embryonic lethality and placenta dysplasia by embryonic day 13.5 (E13.5). (A) Schematic of the mating strategy to generate placental trophoblast‐specific deletion of MNSFβ in mice. (B) Result of in situ hybridization (ISH) showing MNSFβ mRNA (messenger RNA) abundance in the placentas from Control, Het and conditional knockout (cKO) groups at E13.5. Decidua (antisense) and Labyrinth (sense) were included as positive and negative controls, separately. Scale bars indicate 20 μm. (C) Representative views of the uterus in F1 (Cyp19‐Cre^+/−^/ MNSFβ *^loxp^* ^/+^) female mice at E13.5, showing typical embryos and placentas in the Control and cKO groups, respectively. Red arrows represent lethal embryos. (D, E) Statistical analysis of the foetal weight, placental weight in Control, Het and cKO groups at E10.5 (D; *N* = 6) and E13.5 (E; *N* = 12). Data are presented as mean ± SEM, and comparison between groups is carried out with one‐way analysis of variance (ANOVA) with Sidak's correction. *p < 0.05, **, *p* < 0.01

To specifically elucidate the role of MNSFβ in placental trophoblasts, we generated a cKO mouse model targeting the *MNSFβ* gene in trophoblastic lineage using a *Cyp19*‐*Cre* mouse, which has been well accepted as a tool to trigger trophoblast‐specific gene manipulation from E6.5.^13^, ^21^ Intercrossing of *MNSFβ^loxp^* ^/^ *^loxp^* mice with *Cyp19*‐*Cre* mice generated homozygous (*Cyp19*‐*Cre* ^+/−^/*MNSFβ^loxp^* ^/^ *^loxp^*; cKO group) and heterozygous (*Cyp19*‐*Cre* ^+/−^/ *MNSFβ^loxp^* ^/+^; Het group) deficiencies of MNSFβ in multiple subtypes of trophoblasts (Figure 2A). The results of ISH for MNSFβ in placental tissues at E13.5 proved the high efficiency of gene deletion in trophoblasts in the cKO group (Figure 2B).

Our results of genotyping for the born pups revealed no cKO foetus survive to birth, while Het foetuses exhibited a normal birth rate and little obvious abnormality at birth. At E10.5 and E13.5, the ratio of cKO:Het:Control embryos was approximately 1:1:2, indicating that cKO embryos could survive till mid‐gestation (Figure 2C and Table S3). However, the embryonic weight and placental weight of cKO embryos were remarkably lowered at E13.5 compared to those of the Het or Control group, although the decreased tendency was not significant at E10.5 (Figure 2D,E).

The phenotype observations reveal that trophoblast‐specific deficiency in MNSFβ results in retarded embryonic and placental development and subsequent foetal lethality.

### 3.3 Deletion of MNSFβ in the placenta impairs trophoblast cell invasiveness

**FIGURE 3:**
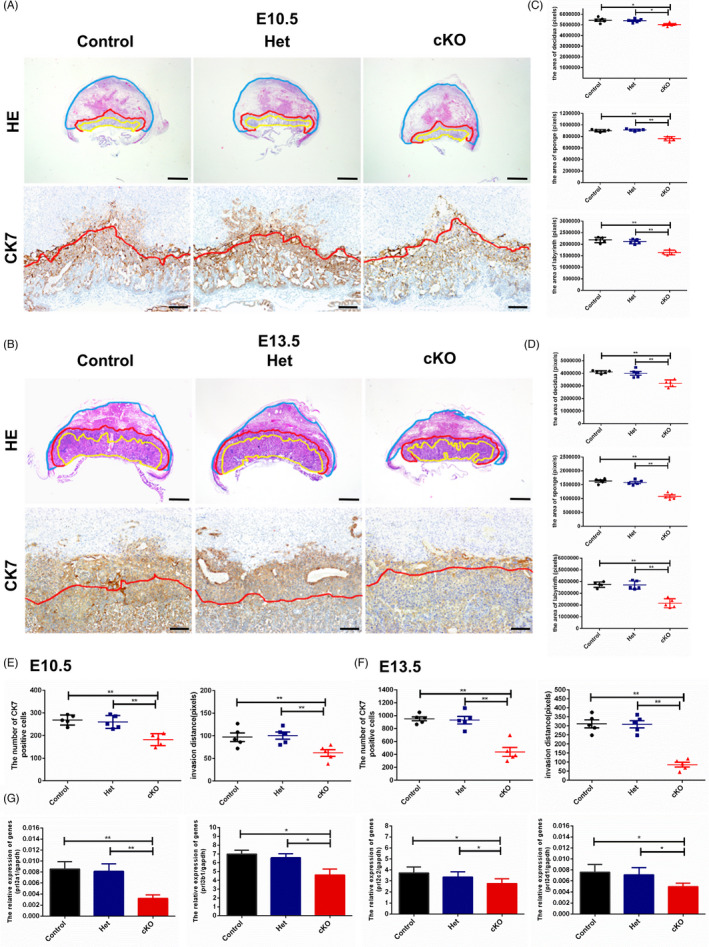
Defect in trophoblastic invasiveness in trophoblast‐specific deletion of monoclonal nonspecific suppressor factor beta (MNSFβ) at embryonic day 10.5 (E10.5) and embryonic day 13.5 (E13.5). (A, B) Typical results of haematoxylin and eosin (HE) and immunohistochemistry (IHC) for cytokeratin 7 (CK7) in placentas from Control, Het and conditional knockout (cKO) groups at E10.5 (A) and E13.5 (B), respectively. Based on the HE and CK7 staining, decidua, sponge zone and labyrinth layers are separately marked by blue, red and yellow solid lines. Scale bars indicate 500 μm (upper panels) and 100 μm (lower panels). (C, D) Statistical analysis of the areas of decidua, sponge zone and labyrinth layers in Control, Het and cKO placentas at E10.5 (C) and E13.5 (D) (*n* = 5 in each group). (E, F) Statistical analysis of the number of trophoblasts that infiltrated into decidua and the invasion distance at E10.5 (E) and E13.5 (F) (*n* = 5 in each group). (G) Statistical results of quantitative real‐time PCR for invasion‐related marker genes (prl3a1, prl3b1, prl2c2 and prl3d1) in the placentas from Control, Het and cKO at E13.5 (*n* = 5 in each group). The experiments are independently repeated for three times, and data are presented as mean ± SEM. Comparison between groups is carried out with one‐way analysis of variance (ANOVA) with Sidak's correction. *p < 0.05, **p < 0.01

We further examined the placental structures at E10.5 and E13.5 in the mice. Histological analysis revealed little difference between the placentas of the Het and Control groups in the area of the three functional layers – Dec, Sp and Lab at E10.5 or E13.5 (Figure 3A,B). Whereas in cKO placentas, the areas of Dec, Sp and Lab at E10.5 were separately reduced to approximately 90%, 85% and 80% of the corresponding Control (Figure 3C), which were more severe at E13.5, being approximately 80%, 65% and 60% of the corresponding Control (Figure 3D).

Immunohistochemistry (IHC) for CK7 was performed to mark trophoblast cells and thus the cell invasiveness was analysed (Figure 3A,B). As shown, both the number of trophoblast cells that invaded into the decidual layer and their invasion distance were markedly declined in cKO placentas, which were approximately half of the Control at E10.5 (Figure 3E) and less than one third of the Control at E13.5 (Figure 3F). In parallel, the results of quantitative real‐time PCR showed a remarkable downregulation of the invasion‐associated marker genes (*prl3a1*, *prl3b1*, *prl2c2 and prl3d1*) in cKO placentas, relative to the Het or Control group (Figure 3G).

These data demonstrate that the impaired trophoblast cell invasion due to MNSFβ deficiency is a primary cellular defect in association with retarded embryonic and placental development.

### 3.4 MNSFβ can directly bind IGF2BP2 in trophoblast cell

Previous studies have indicated the intracellular functions of MNSFβ primarily through binding other proteins. To explore how MNSFβ regulates trophoblast cell invasion, we screened proteins that can potentially interact with MNSFβ in a trophoblast cell line, JEG3, by using the GST pull‐down and Orbitrap Elite Mass Spectrometer (OEMS) (Table S4).

**FIGURE 4:**
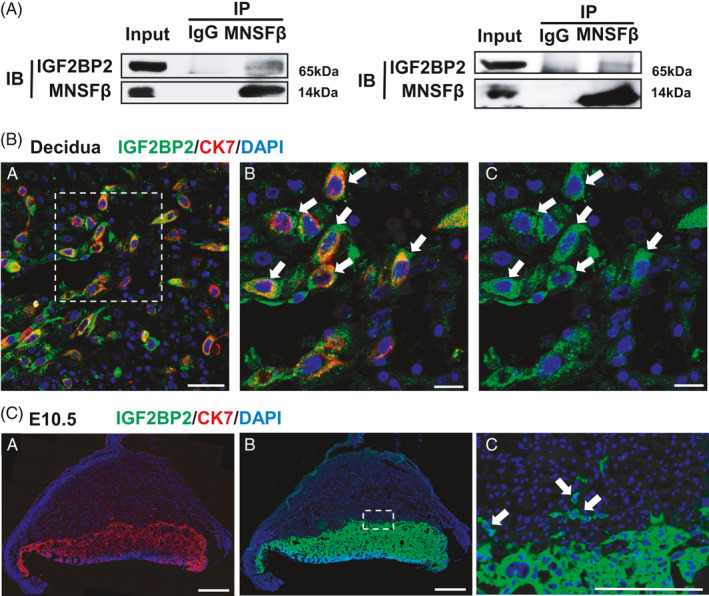
Monoclonal nonspecific suppressor factor beta (MNSFβ) directly interacts with insulin‐like growth factor 2 mRNA binding protein 2 (IGF2BP2) in human trophoblast cell. (A) Typical result of immunoprecipitation (IP) of MNSFβ to IGF2BP2 in JEG3 (left) and human trophoblast stem cell (hTSC) (right) cells. (B) Typical results of the immunofluorescent staining of IGF2BP2 (green) and cytokeratin 7 (CK7) (red) in human decidua at weeks 7–9. (b and c), Enlargement of the area, as indicated in panel (a). (C) Immunofluorescent staining of IGF2BP2 (green) and CK7 (red) in mouse placentas at embryonic day 10.5 (E10.5). (c), Enlargement of the area, as indicated in panel (b). White arrows represent human extravillous trophoblast (EVT) cells or the trophoblasts that invaded into decidua of mouse placenta. Scale bars indicate 50 μm in (a) of (B), 20 μm in (b‐c) of (B), 600 μm in (a‐b) of (C) or 100 μm in (c) of (C)

Among the 24 candidates as shown in Table S4, IGF2BP2 has been reported as an important intracellular factor for female fertility and trophoblast invasion.^22^, ^23^ We carried out IP analysis in both JEG3 cells and human trophoblast stem cells (hTSCs) and proved the direct binding of MNSFβ to IGF2BP2 (Figure 4A). Immunofluorescent staining for IGF2BP2 was then performed to clarify its distribution at the foeto‐maternal interface of human and mouse. In human decidual tissues at early gestation, immunofluorescent signals were primarily observed in EVT cells (Figure 4B). At mouse foeto‐maternal interface at E10.5, IGF2BP2 staining was found in multiple subtypes of trophoblasts, including the trophoblasts that invaded into the decidual tissue (Figure 4C). These observations suggest the probable involvement of IGF2BP2 in MNSFβ‐regulated trophoblastic invasiveness.

### 3.5 MNSFβ binds with IGF2BP2 to protect its degradation by ubiquitin‐proteasome in trophoblast cell

**FIGURE 5:**
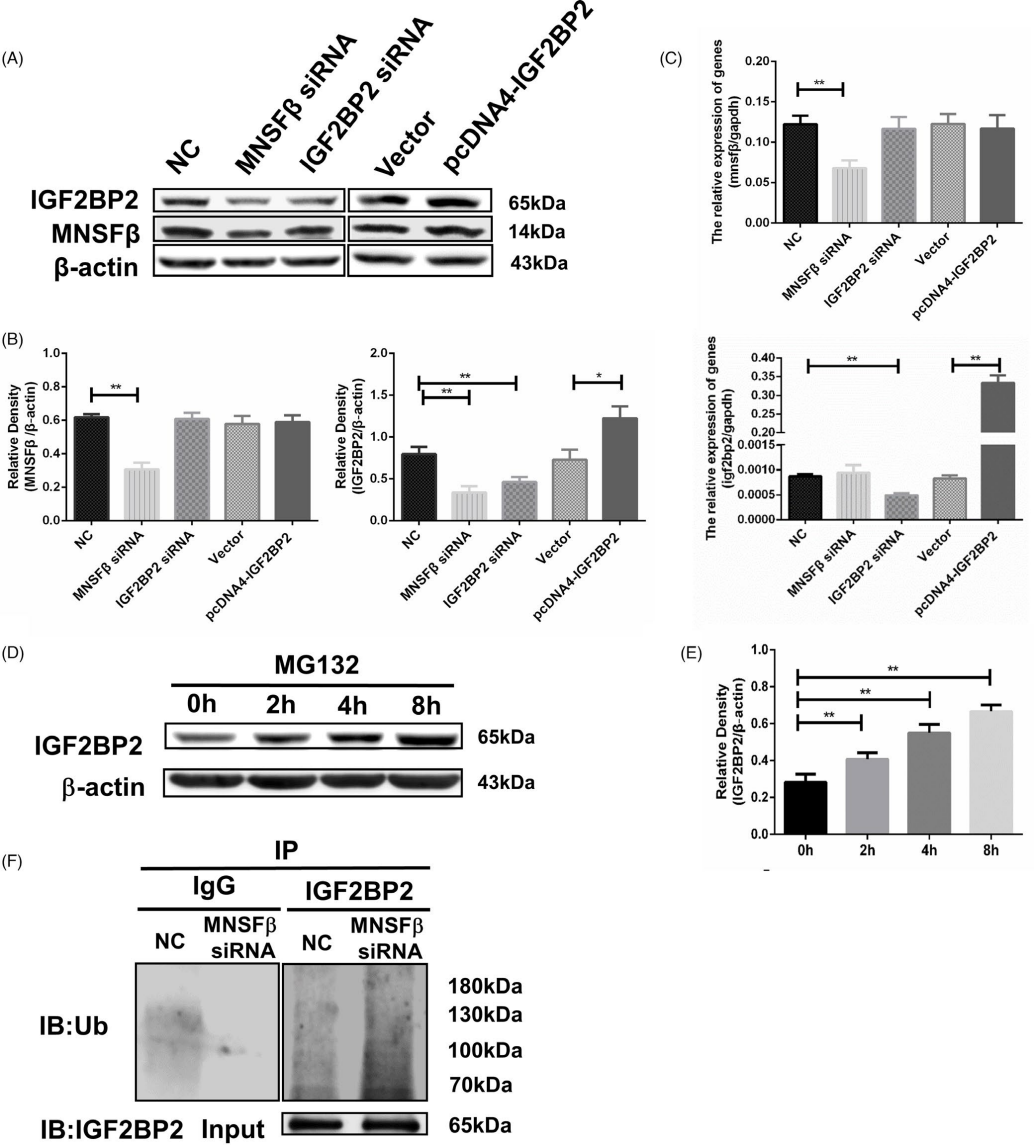
Interaction between monoclonal nonspecific suppressor factor beta (MNSFβ) and insulin‐like growth factor 2 mRNA‐binding protein 2 (IGF2BP2) in JEG3 cells. (A, B, C) Typical results (A) and statistical analysis (B) of immunoblotting, and statistical analysis of real‐time polymerase chain reaction (RT‐PCR) (C) for MNSFβ and IGF2BP2 in JEG3 cells following transfection with MNSFβ siRNA (small interfering RNA) (80 nmol/L), IGF2BP2 siRNA (80 nmol/L), scrambled siRNA (NC, 80 nmol/L), plasmid vector (Vector) or IGF2BP2‐overexpressing construct (pcDNA4‐IGF2BP2). (D, E) Typical results (D) and statistical analysis (E) of immunoblotting for IGF2BP2 in JEG3 cells treated with carbobenzoxy‐Leu‐Leu‐leucinal (MG132) (10 μM) for different time periods. (F) Immunoprecipitation with antibody against IGF2BP2 or immunoglobulin G (IgG), followed by immunoblotting for ubiquitin (Ub) or IGF2BP2 in JEG3 cells transfected with MNSFβ siRNA or scrambled siRNA (NC). Data are presented as mean ± SEM based on three independently repeated experiments, and comparison between groups is carried out with Student's *t*‐test. *p < 0.05, **p < 0.01

Interestingly, knockdown of MNSFβ in JEG3 cells using specific siRNA did not alter the mRNA level of IGF2BP2, but led to a markedly reduced IGF2BP2 protein level, being less than half of that in the NC group in which scramble siRNA was transfected (Figure 5A‐C). On the contrary, knockdown or overexpression of IGF2BP2 had little influence on MNSFβ expression (Figure 5A‐C). These data indicate that the direct binding of MNSFβ to IGF2BP2 may increase the stability of IGF2BP2 protein in trophoblasts.

In JEG3 cells, we found a gradually elevated IGF2BP2 protein level upon the treatment of the proteasome inhibitor carbobenzoxy‐Leu‐Leu‐leucinal (MG132), whereas the autophagy inhibitor 3‐methyladenine (3‐MA) had little influence (Figure 5D,E and Figure S1). In addition, knocking‐down of MNSFβ significantly increased the ubiquitination level of IGF2BP2 in JEG3 cells (Figure 5F).

These data demonstrate that MNSFβ binding with IGF2BP2 protects the degradation of IGF2BP2 by the ubiquitin‐proteasome pathway.

### 3.6 MNSFβ promotes trophoblast cell invasion via interaction with IGF2BP2

**FIGURE 6:**
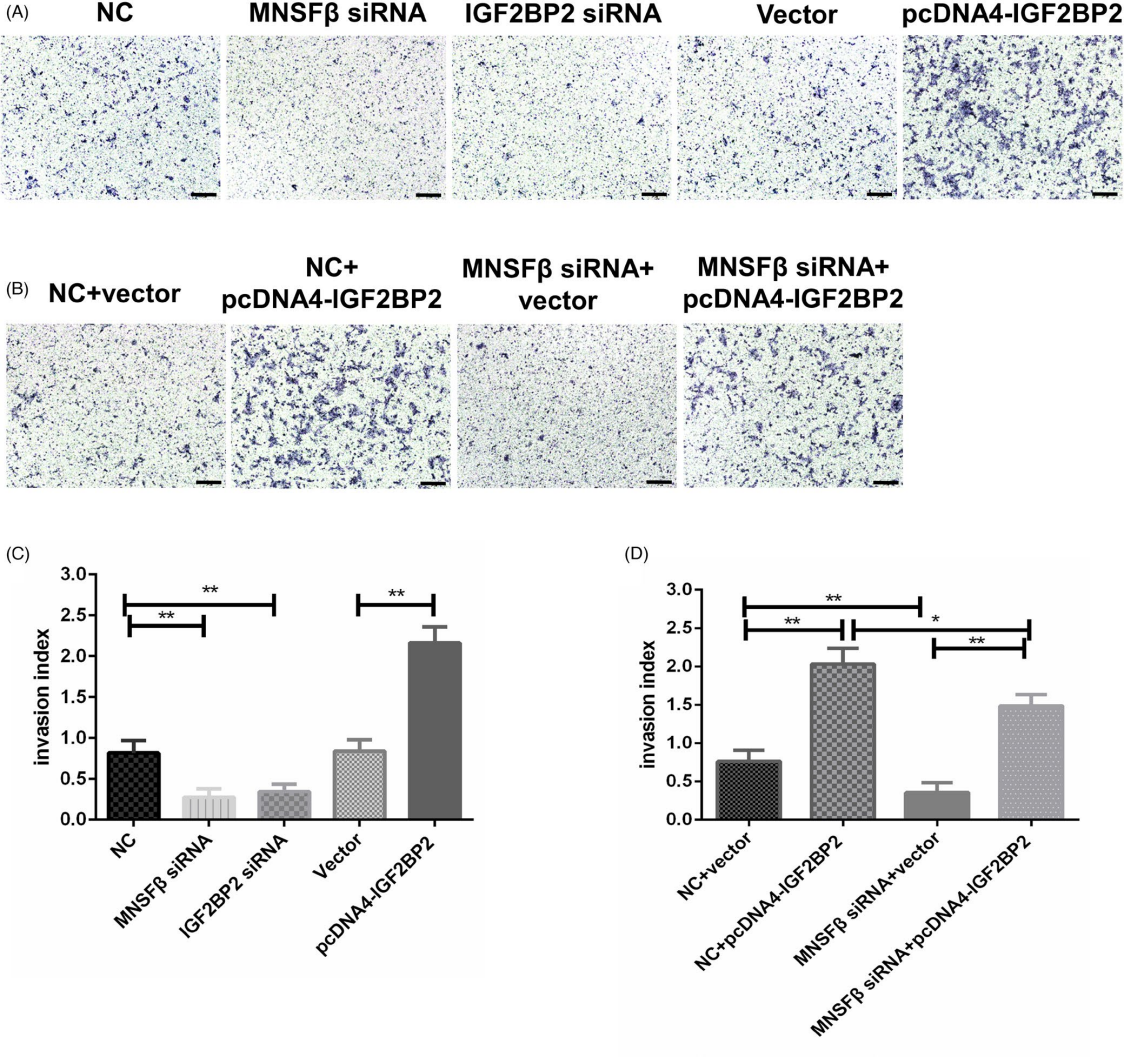
Monoclonal nonspecific suppressor factor beta (MNSFβ) fosters cell invasion through interaction with insulin‐like growth factor 2 mRNA‐binding protein 2 (IGF2BP2) in JEG3 cells. (A, C) Typical results (A) and statistical analysis (C) of Transwell insert assay in JEG3 cells following transfection with MNSFβ siRNA (small interfering RNA), IGF2BP2 siRNA, scrambled siRNA (NC), plasmid vector (Vector), or IGF2BP2‐overexpressing construct (pcDNA4‐IGF2BP2). (B, D) Typical results (B) and statistical analysis (D) of the Transwell insert assay in JEG3 cells following transfection with MNSFβ siRNA together with or without IGF2BP2‐overexpressing construct (pcDNA4‐IGF2BP2). Scale bars indicate 100 μm. In each group, 10 views were randomly selected and the invaded cells were quantified. Data are presented as mean ± SEM based on three independently repeated experiments, and comparison between groups is carried out with Student's *t*‐test. *p < 0.05, **, *p* < 0.01

To interpret whether the interaction of MNSFβ with IGF2BP2 regulates trophoblast cell invasion, we carried out the Transwell insert invasion assay in JEG3 and HTR8/SVneo cells that were subjected to knockdown or overexpression of MNSFβ or IGF2BP2. As shown in Figure 6, Figure S2 and S3, downregulation of either MNSFβ or IGF2BP2 with specific siRNA led to evidently reduced invasive capacity, while the overexpression of IGF2BP2 greatly enhanced cell invasiveness (Figure 6A,C and Figure S2A,C) in JEG3 and HTR8/SVneo cells. What's more, the invasion‐inhibiting effect of MNSFβ siRNA could be partially, while significantly, reversed by increasing IGF2BP2 expression (Figure 6B,D and Figure S2B,D). The data reveal that MNSFβ promotes trophoblast cell invasion via, at least in part, directly interacting with IGF2BP2.

### 3.7 Downregulation of IGF2BP2 is associated with repressed MNSFβ in the placentas derived from cKO mice and severe preeclamptic (PE) patients

**FIGURE 7:**
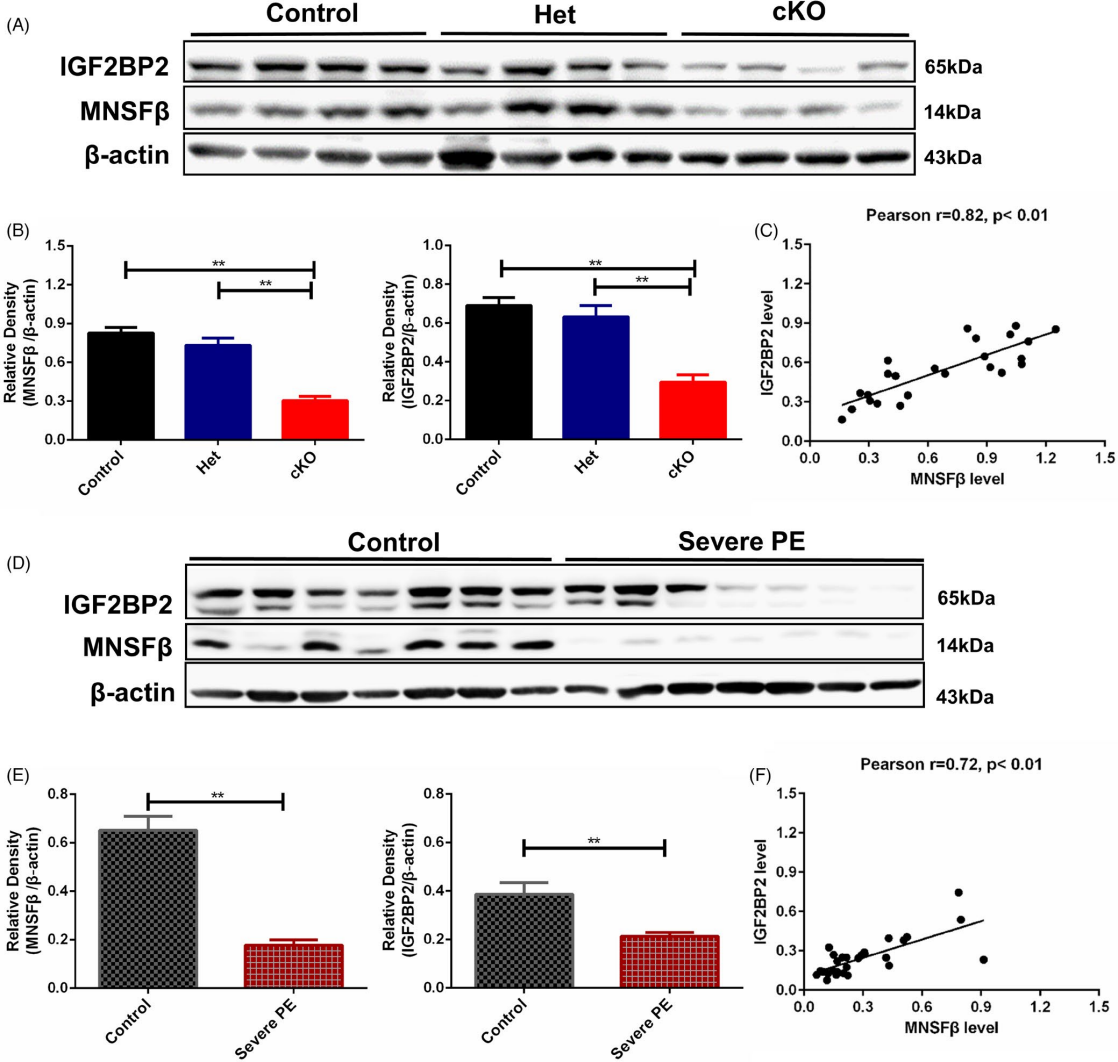
Expressions of monoclonal nonspecific suppressor factor beta (MNSFβ) and insulin‐like growth factor 2 mRNA‐binding protein 2 (IGF2BP2) in the placentas derived from MNSFβ‐deletion mice and preeclamptic (PE) patients. (A‐B) Typical results (A) and statistical analysis (B) of immunoblotting for MNSFβ and IGF2BP2 in the placentas from Control, Het and conditional knockout (cKO) mice at embryonic day 13.5 (E13.5) (*n* = 8 in each group). (C) Correlation analysis between MNSFβ and IGF2BP2 in cKO placentas at E13.5. (D‐E) Typical results (D) and statistical analysis (E) of immunoblotting for MNSFβ and IGF2BP2 in the placentas from unexplained preterm labour control (PTL) and severe PE patients (*n* = 7 in each group). (F) Correlation analysis between MNSFβ and IGF2BP2 in the placentas of PTL and severe PE. Data are presented as mean ±SEM based on three independently repeated experiments, and comparison between groups is carried out with Student's *t*‐test or one‐way analysis of variance (ANOVA) with Sidak's correction. *p < 0.05, **p < 0.01

Based on the above evidence, we measured the protein levels of IGF2BP2 and MNSFβ in the placentas from the MNSFβ‐cKO mice at E13.5. Because MNSFβ was widely expressed in multiple cell types at the foeto‐maternal interface (as shown in Figure 1B,C), the results of immunoblotting in the placental tissues revealed an approximately 70% decrease in the MNSFβ‐cKO group compared with Control or Het groups (Figure 7A,B). The level of IGF2BP2 protein in these cKO placentas was reduced by nearly 60% relative to Control or Het groups (Figure 7A,B). Statistical analysis demonstrated a tight association between the protein level of MNSFβ and IGF2BP2 in cKO placentas (Figure 7C). In parallel, the ubiquitination level of IGF2BP2 in cKO placentas at E13.5 obviously elevated compared to the Control group (Figure S4A).

It has been well accepted that compromised trophoblast cell invasion and subsequent insufficient spiral artery remodelling are predominant pathological changes in PE placenta, especially in those cases with severe symptoms. We thus examined the protein levels of MNSFβ and IGF2BP2 in the placentas from severe PE patients. Considering most of the severe PE placentas were delivered at earlier than the 37th gestational week, we collected placentas from unexplained PTL as the gestational‐week‐matched control.^14^ As expected, the levels of MNSFβ and IGF2BP2 were significantly lower in PE placentas, being approximately 40% and 35% of the corresponding PTL control, respectively (Figure 7D,E). Statistical analysis showed a tight association between the protein level of MNSFβ and IGF2BP2 in PE placentas (Figure 7F). As expected, the ubiquitination level of IGF2BP2 obviously increased in PE placentas compared to PTL control (Figure S4B).

## 4 DISCUSSION

A recent systematic study in mice with embryonic‐lethal mutations demonstrates that a considerable number of embryonic defects may result from defective placentation, robustly revealing the vital role of a healthy placenta for embryonic development.^3^ In this study, the mice with trophoblastic deficiency of MNSFβ exhibited a similar phenotype of embryonic lethality with that of cKO mice.^11^ As stated, the deficiency of MNSFβ in trophoblasts from E6.5 causes an apparently limited trophoblast cell invasiveness at E10.5, leading to great retardation in embryonic and placental development and eventually no surviving foetus. MNSFβ is widely expressed in multiple cell types at the foeto‐maternal interface, the early placental dysplasia in trophoblast‐specific deletion of MNSFβ strongly indicating the critical roles of this molecule for early differentiation of PHTs and thus for pregnancy maintenance.

The appropriate trophoblast differentiation towards invasive pathway is important to anchor the embryo into uterine wall and to build up proper blood perfusion into the foeto‐maternal interface. Limited invasiveness has been recognized as the major pathological factor for various adverse pregnancy outcomes, including PE, foetal growth restriction or early foetal loss.^24^, ^25^, ^26^, ^27^ IGF2BP2 has been found to stimulate invasion and migration of trophoblast cell, and its protein level is significantly lower in severe PE placentas.^22^ Our findings substantiate the role of IGF2BP2 in controlling trophoblastic fate towards invasive pathway, and clearly reveals the vital role of MNSFβ to stabilize IGF2BP2 and thus reinforce its invasion‐enhancing effect.

MNSFβ has long been known as a non‐antigen‐specific immunosuppressive factor. It was first found to be a lymphokine produced by murine T cell hybridoma which can inhibit the secretion of immunoglobulin (Ig) from LPS‐induced mononuclear cells.^28^ Our previous observation that MNSFβ acts in a paracrine manner to promote interleukin‐4 (IL‐4) while inhibiting tumour necrosis factor‐alpha (TNF‐α) production in mouse lymphocytes also indicates its property as a lymphokine.^7^ Later studies reveal its participation in modulating apoptosis and phagocytosis of macrophage through intracellularly interacting with other proteins. For instance, MNSFβ conjugates to Bcl‐G and strongly promotes lipopolysaccharide (LPS)/interferon gamma (IFNγ)‐triggered apoptosis in mouse macrophage by downregulating the extracellular signal‐regulated kinase (ERK)/activator protein 1 (AP‐1) signalling cascade and thus cyclooxygenase‐2 (Cox‐2) activation.^29^ MNSFβ–endophilin II complex can inhibit phagocytosis of macrophages by inhibiting the signalling upstream of IKK activation.^30^ This information indicates a wide spectrum of MNSFβ's interacting targets and may act more than an immunosuppressive factor. Our preliminary experiments demonstrated a rather low level of secreted MNSFβ in the primary culture of human PHTs and JEG3 cells. In addition, the invasion‐promoting effect of the condition media (CM) from PHT cells could hardly be blocked by pre‐treating CM with anti‐MNSFβ neutralizing antibody. Thus, we assumed that MNSFβ regulated trophoblast invasion in an intracellular manner, but not in secreted form. On the other hand, our study in human trophoblast cells demonstrated little influence of MNSFβ on cell apoptotic activity, suggesting the distinct working mechanisms of MNSFβ in PHTs from those in macrophages.^12^ As indeed, our results of GST pull‐down in human trophoblast cells identified several proteins that potentially bind to MNSFβ (Table S4), which were not similar to those found in macrophages. Such a difference also indicated that the effect of MNSFβ may largely depend on the intracellular microenvironment.

Here in our study, in vitro and in vivo experiments proved a direct effect of MNSFβ on trophoblast cell invasion, partly through an intracellular interaction with IGF2BP2. However, it is notable that among the candidates that potentially bind to MNSFβ, ribosomal protein S3A (RPS3A) was reported to promote the biological processes related to tumourigenesis, metastasis and immunosuppression in hepatocellular carcinoma patients,^31^ human zinc finger RNA‐binding protein (ZFR) repressed the interferon response by preventing aberrant splicing and nonsense‐mediated decay of histone variant macroH2A1/H2AFY mRNAs to regulate macrophage differentiation.^32^ These evidences suggest the possibility of MNSFβ to regulate the production of immune factors in human trophoblasts. Therefore, embryonic lethality in MNSFβ‐cKO mice may also result from the compromised produce of immune‐regulating factors from MNSFβ‐deficient trophoblasts which contributes to the imbalance of immune status at the maternal–foetal interface. Further exploration of the interactions between MNSFβ and other proteins will warrant an in‐depth understanding of how MNSFβ controls the trophoblast cell fate and pregnancy outcomes.

Our results in MG132‐treated or MNSFβ‐knockdown JEG3 cells, and the placentas from MNSFβ‐cKO mice or severe PE patients strongly indicated the protection of IGF2BP2 from ubiquitin‐proteasome degradation by MNSFβ in trophoblasts; whereas the underlying mechanism of IGF2BP2 stabilization by MNSFβ remains unclear. Previous evidences have shown that MNSFβ can covalently attach to specific target proteins with a linkage between the C‐terminal G74 and certain lysines, such as K110 in Bcl‐G, K294 in endophilin II, K481 in heat shock protein 60 (HSP60) and K72 in formate dehydrogenase (FDH).^30^, ^33^, ^34^, ^35^ K139 in IGF2BP2 has been the predominant site for its ubiquitination.^36^ We thus assume that the covalent binding between G74 in MNSFβ with K139 in IGF2BP2 may be the recognition site to protect the degradation of IGF2BP2.

Interestingly, our results showed that the areas of all three functional layers, decidua, sponge zone and labyrinth, significantly decreased in MNSFβ‐cKO mice, indicating that the embryonic lethality, placental and foetal maldevelopment in MNSFβ‐deficient mice may not be solely caused by an impaired EVT invasion, but may also involve compromised trophoblast syncytialization and thus placental haemodynamics or spongiotrophoblast differentiation. On the other hand, considering the extensive expression of MNSFβ in diverse cell types at the foeto‐maternal interface and its lymphokine property, there is a possibility that MNSFβ from other cells may, to a certain extent, compensate its deletion in trophoblasts, leading to a relatively late appearance of the placental phenotypes in cKO mice. Further investigation using models such as trophoblast subtype‐specific deletion or tetraploid compensation is needed to address these issues.

In general, our findings reveal the crucial role of MNSFβ in modulating trophoblast differentiation at the early stage of gestation. Particularly, it affects trophoblastic invasiveness by binding and stabilizing IGF2BP2. The study also indicates the participation of functionally deficient MNSFβ in the pathogenesis of pregnancy disease such as PE.

## CONFLICT OF INTERESTS

The authors declare no competing or financial interests.

## AUTHOR CONTRIBUTIONS

Q.Y. and Y.M. performed experiments, analysed the data and drafted the manuscript. X.S. constructed plasmids and drafted the manuscript. Y.L. and W.J. performed mass spectrometry experiments and helped in mouse maintenance. X.Y. cultured hTSC. Y.L. helped in analysing clinical data. L.Y and W.G performed GST pull‐down assay. J.W. and Y.W. designed and supervised the study, interpreted the data and revised the manuscript. All authors commented on the manuscript.

## Supporting information

Supplementary Material

## Data Availability

The data of this study are available from the corresponding author upon reasonable request.

## References

[1] Anin SA , Vince G , Quenby S . Trophoblast invasion. Hum Fertil (Camb). 2004;7(3):169‐174. 15590570 10.1080/14647270400006911

[2] Rossant J , Cross JC . Placental development: lessons from mouse mutants. Nat Rev Genet. 2001;2(7):538‐548. 11433360 10.1038/35080570

[3] Perez‐Garcia V , Fineberg E , Wilson R , et al. Placentation defects are highly prevalent in embryonic lethal mouse mutants. Nature. 2018;555(7697):463‐468. 29539633 10.1038/nature26002 PMC5866719

[4] Reynolds LP , Redmer DA . Angiogenesis in the placenta. Biol Reprod. 2001;64(4):1033‐1040. 11259247 10.1095/biolreprod64.4.1033

[5] Red‐Horse K , Zhou Y , Genbacev O , et al. Trophoblast differentiation during embryo implantation and formation of the maternal‐fetal interface. J Clin Investig. 2004;114(6):744‐754. 15372095 10.1172/JCI22991 PMC516273

[6] Nie GY , Li Y , Hampton AL , Salamonsen LA , Clements JA , Findlay JK . Identification of monoclonal nonspecific suppressor factor beta (mNSFbeta) as one of the genes differentially expressed at implantation sites compared to interimplantation sites in the mouse uterus. Mol Reprod Dev. 2000;55(4):351‐363. 10694741 10.1002/(SICI)1098-2795(200004)55:4<351::AID-MRD1>3.0.CO;2-L

[7] He Y , Sun Z , Shi Y , Jiang Y , Jia Z , Du Y , Salamonsen L , Li Z , Wang J . Immunosuppressive Factor MNSFβ Regulates Cytokine Secretion by Mouse Lymphocytes and Is Involved in Interactions between the Mouse Embryo and Endometrial Cells In Vitro. ISRN Immunology. 2011;2011:1‐11. 10.5402/2011/186541

[8] Kas K , Michiels L , Merregaert J . Genomic structure and expression of the human fau gene: encoding the ribosomal protein S30 fused to a ubiquitin‐like protein. Biochem Biophys Res Comm. 1992;187(2):927‐933. 1326960 10.1016/0006-291x(92)91286-y

[9] Nakamura M , Xavier RM , Tanigawa Y . Monoclonal nonspecific suppressor factor beta inhibits interleukin‐4 secretion by a type‐2 helper T cell clone. Eur J Immunol. 1995;25(8):2417‐2419. 7664805 10.1002/eji.1830250844

[10] Nakamura M , Tanigawa Y . Ubiquitin‐like polypeptide inhibits cAMP‐induced p38 MAPK activation in Th2 cells. Immunobiology. 2004;208(5):439‐444. 15124858 10.1078/0171-2985-00291

[11] Gu Y , He Y , Zhang X , et al. Deficiency of monoclonal non‐specific suppressor factor beta (MNSFB) promotes pregnancy loss in mice. Mol Reprod Dev. 2015;82(6):475‐488. 26031240 10.1002/mrd.22495

[12] Wang N , Yang Q , Gu Y , et al. MNSFbeta promotes the proliferation and migration of human extravillous trophoblast cells and the villus expression level of MNSFbeta is decreased in recurrent miscarriage patients. Gynecol Obstet Invest. 2020;1‐13. 33326956 10.1159/000506309

[13] Wenzel PL , Leone G . Expression of Cre recombinase in early diploid trophoblast cells of the mouse placenta. Genesis. 2007;45(3):129‐134. 17299749 10.1002/dvg.20276

[14] Zhou Y , Bianco K , Huang L , et al. Comparative analysis of maternal‐fetal interface in preeclampsia and preterm labor. Cell Tissue Res. 2007;329(3):559‐569. 17549520 10.1007/s00441-007-0428-0

[15] Mol BWJ , Roberts CT , Thangaratinam S , Magee LA , de Groot CJM , Hofmeyr GJ . Pre‐eclampsia. Lancet. 2016;387(10022):999‐1011. 26342729 10.1016/S0140-6736(15)00070-7

[16] Okae H , Toh H , Sato T , et al. Derivation of human trophoblast stem cells. Cell Stem Cell. 2018;22(1):50‐63 e6. 29249463 10.1016/j.stem.2017.11.004

[17] Gu Y , Shi Y , Yang Q , et al. miR‐3074‐5p promotes the apoptosis but inhibits the invasiveness of human extravillous trophoblast‐derived HTR8/SVneo cells in vitro. Reprod Sci. 2018;25(5):690‐699. 28826362 10.1177/1933719117725823

[18] Yang Q , Gu WW , Gu Y , et al. Association of the peripheral blood levels of circulating microRNAs with both recurrent miscarriage and the outcomes of embryo transfer in an in vitro fertilization process. J Transl Med. 2018;16(1):186. 29973278 10.1186/s12967-018-1556-x PMC6032771

[19] Livak KJ , Schmittgen TD . Analysis of relative gene expression data using real‐time quantitative PCR and the 2(‐Delta Delta C(T)) Method. Methods. 2001;25(4):402‐408. 11846609 10.1006/meth.2001.1262

[20] Ma L , Li G , Cao G , et al. dNK cells facilitate the interaction between trophoblastic and endothelial cells via VEGF‐C and HGF. Immunol Cell Biol. 2017;95(8):695‐704. 28653669 10.1038/icb.2017.45

[21] Wenzel PL , Wu L , de Bruin A , et al. Rb is critical in a mammalian tissue stem cell population. Genes Dev. 2007;21(1):85‐97. 17210791 10.1101/gad.1485307 PMC1759903

[22] Wu L , Song WY , Xie Y , et al. miR‐181a‐5p suppresses invasion and migration of HTR‐8/SVneo cells by directly targeting IGF2BP2. Cell Death Dis. 2018;9(2):16. 29339719 10.1038/s41419-017-0045-0 PMC5833820

[23] Liu HB , Muhammad T , Guo Y , et al. RNA‐binding protein IGF2BP2/IMP2 is a critical maternal activator in early zygotic genome activation. Adv Sci (Weinh). 2019;6(15):1900295. 31406667 10.1002/advs.201900295 PMC6685478

[24] Abbas Y , Turco MY , Burton GJ , Moffett A . Investigation of human trophoblast invasion in vitro. Hum Reprod Update. 2020;26(4):501‐513. 32441309 10.1093/humupd/dmaa017 PMC7473396

[25] Moffett A , Loke C . Immunology of placentation in eutherian mammals. Nat Rev Immunol. 2006;6(8):584‐594. 16868549 10.1038/nri1897

[26] Pijnenborg R , Vercruysse L , Hanssens M . The uterine spiral arteries in human pregnancy: facts and controversies. Placenta. 2006;27(9–10):939‐958. 16490251 10.1016/j.placenta.2005.12.006

[27] Redman CW , Sargent IL . Latest advances in understanding preeclampsia. Science. 2005;308(5728):1592‐1594. 15947178 10.1126/science.1111726

[28] Nakamura M , Ogawa H , Tsunematsu T . Isolation and characterization of a monoclonal nonspecific suppressor factor (MNSF) produced by a T cell hybridoma. J Immunol. 1986;136(8):2904‐2909. 2420877

[29] Watanabe J , Nakagawa M , Watanabe N , Nakamura M . Ubiquitin‐like protein MNSFbeta covalently binds to Bcl‐G and enhances lipopolysaccharide/interferon gamma‐induced apoptosis in macrophages. FEBS J. 2013;280(5):1281‐1293. 23298187 10.1111/febs.12120

[30] Nakamura M , Watanabe N . Ubiquitin‐like protein MNSFbeta/endophilin II complex regulates Dectin‐1‐mediated phagocytosis and inflammatory responses in macrophages. Biochem Biophys Res Comm. 2010;401(2):257‐261. 20849826 10.1016/j.bbrc.2010.09.045

[31] Zhou C , Weng J , Liu C , et al. High RPS3A expression correlates with low tumor immune cell infiltration and unfavorable prognosis in hepatocellular carcinoma patients. Am J Cancer Res. 2020;10(9):2768‐2784. 33042616 PMC7539769

[32] Haque N , Ouda R , Chen C , Ozato K , Hogg JR . ZFR coordinates crosstalk between RNA decay and transcription in innate immunity. Nat Commun. 2018;9(1):1145. 29559679 10.1038/s41467-018-03326-5 PMC5861047

[33] Nakamura M , Tanigawa Y . Characterization of ubiquitin‐like polypeptide acceptor protein, a novel pro‐apoptotic member of the Bcl2 family. Eur J Biochem. 2003;270(20):4052‐4058. 14519116 10.1046/j.1432-1033.2003.03790.x

[34] Nakamura M , Watanabe N , Notsu K . Ubiquitin‐like protein MNSFbeta covalently binds to cytosolic 10‐formyltetrahydrofolate dehydrogenase and regulates thymocyte function. Biochem Biophys Res Comm. 2015;464(4):1096‐1100. 26192119 10.1016/j.bbrc.2015.07.083

[35] Nakamura M , Notsu K , Nakagawa M . Heat shock protein 60 negatively regulates the biological functions of ubiquitin‐like protein MNSFbeta in macrophages. Mol Cell Biochem. 2019;456(1–2):29‐39. 30710197 10.1007/s11010-018-3487-5

[36] Wang Y , Lu JH , Wu QN , et al. LncRNA LINRIS stabilizes IGF2BP2 and promotes the aerobic glycolysis in colorectal cancer. Mol Cancer. 2019;18(1):174. 31791342 10.1186/s12943-019-1105-0 PMC6886219

