# Supplementary material for: MNSFβ regulates placental development by conjugating IGF2BP2 to enhance trophoblast cell invasiveness
Source: Cell Prolif. 2021 Oct 20;54(12):e13145. doi: 10.1111/cpr.13145 (PMC8666274; doi:10.1111/cpr.13145)

**Table S1. Clinical characteristics of the early pregnant women （gestational weeks 7-9）enrolled in this study**

| **Characteristics** | **Sample 1#** | **Sample 2#** |
| --- | --- | --- |
| Maternal age(year) | 30 | 32 |
| Childbearing history | 0 | 1 |
| Miscarriage history | 0 | 0 |
| Gestational week(w) | 8 | 9 |

**Table S2. Clinical characteristics of the women at gestational weeks 30-36 enrolled in this study**

| **Characteristics** | **Control(n=7)** | **Severe PE(n=7)** |
| --- | --- | --- |
| Maternal age(year) | 30.2±1.8 | 30.7±1.3 |
| BMI(kg/m^2^) | 22.6±2.8 | 23.1±2.4 |
| SBP(mmHg) | 108.7±7.2 | 172.2±10.3* |
| DBP(mmHg) | 72.1±5.9 | 113.4±7.6* |
| 24h proteinuria(g/24 h) | ND | 5.6±1.8 |
| Gestational week(w) | 31.5±2.9 | 32.1±3.4 |

ND, not determined. PE, preeclampsia. BMI, Body-Mass Index. SBP, Systolic Blood Pressure. DBP, Diastolic Blood Pressure. Data are expressed as the Mean ± SEM and compared by one-way ANOVA with Sidak’s correction. *, P < 0.05 (compared with Control).

**Table S3. Number and percentage of the embryos in F1 breeder mice with MNSFβ-conditional knockout at E10.5 and E13.5.**

|  | **Control** | | **Het** | | **cKO** | |
| --- | --- | --- | --- | --- | --- | --- |
|  | E10.5 | E13.5 | E10.5 | E13.5 | E10.5 | E13.5 |
| Number | 26 | 53 | 11 | 29 | 12 | 31 |
| Percentage (%) | 53.1 | 46.9 | 22.4 | 25.7 | 24.5 | 27.4 |

**Table S4. List of potential binding candidates of MNSFβ in JEG3 cells that were identified by GST pull down and IP-OEMS.**

| **Gene names** | **LFQ intensity GST** | **LFQ intensity GST-MNSFβ** | **Σ# PSMs**  **IP** | **Σ# PSMs**  **IP-MNSFβ** |
| --- | --- | --- | --- | --- |
| **RPL7** | 0 | 25568000 | 30 | 45 |
| **PABPC1** | 0 | 140140000 | 67 | 110 |
| **MYO6** | 0 | 13745000 | 3 | 6 |
| **FAM120A** | 0 | 15322000 | 1 | 4 |
| **NPM1** | 15476000 | 1525800000 | 14 | 35 |
| **RPS23** | 0 | 1106100000 | 3 | 5 |
| **SF3B3** | 0 | 122530000 | 21 | 37 |
| **RPL14** | 0 | 13550000 | 5 | 18 |
| **IGF2BP2** | 0 | 40898000 | 11 | 19 |
| **RBMX** | 0 | 79097000 | 21 | 36 |
| **KRT1** | 0 | 26548000 | 425 | 578 |
| **H2AFY** | 0 | 10202000 | 6 | 8 |
| **KRT6B** | 0 | 100010000 | 0 | 122 |
| **SLC25A5** | 0 | 50409000 | 4 | 23 |
| **RPS12** | 0 | 452920000 | 2 | 4 |
| **HNRNPA3** | 0 | 55268000 | 6 | 12 |
| **RPS16** | 0 | 1922200000 | 14 | 22 |
| **HNRPR** | 0 | 8223100 | 21 | 33 |
| **EBNA1BP2** | 0 | 11560000 | 1 | 5 |
| **RPS3A** | 0 | 1508400000 | 9 | 17 |
| **LARP1** | 0 | 110420000 | 2 | 12 |
| **H1FX** | 0 | 62172000 | 1 | 3 |
| **DDX21** | 0 | 117640000 | 4 | 9 |
| **SUPT16H** | 0 | 5300400 | 0 | 8 |

**Figure S1.** **Immunoblotting (A) and statistical analysis (B) for IGF2BP2 in JEG3 cells treated with autophagy inhibitor 3-MA for 4h, 12h.**  Data are presented as Mean ± SEM based on the results of three independently repeated experiments. Comparison between groups is carried out with Student's t-test.


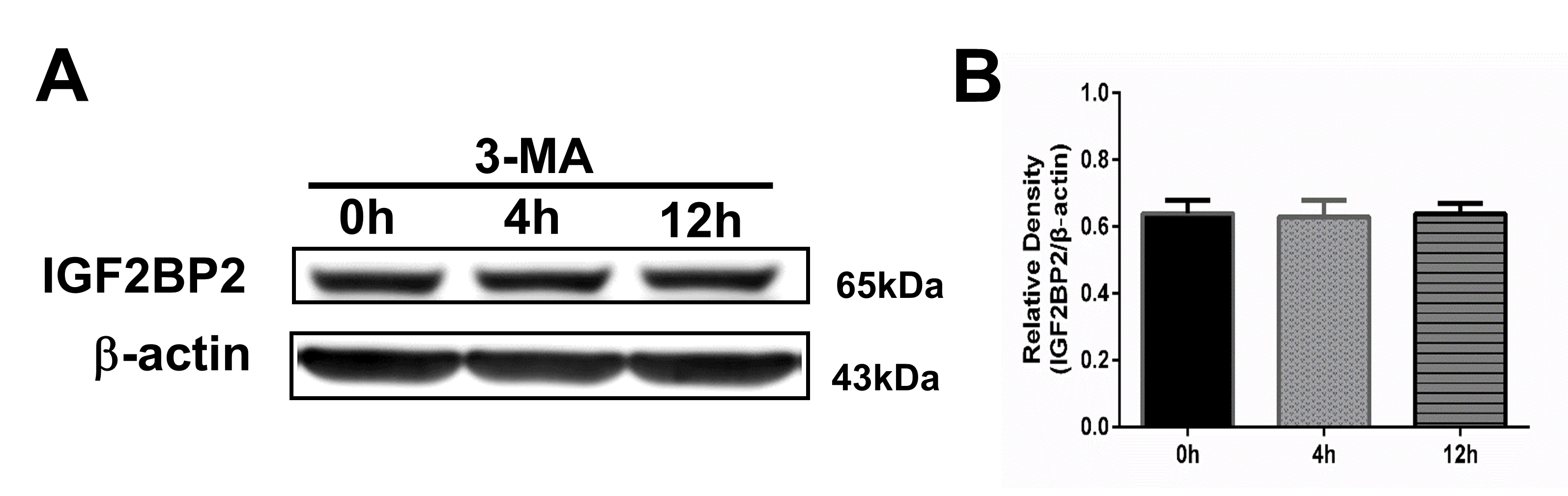


**Figure S2. MNSFβ fosters cell invasion through interaction with IGF2BP2 in HTR8/SVneo cells.** (A, C) Typical results (A) and statistical analysis (C) of Transwell insert assay in HTR8/SVneo cells following transfection with MNSFβ siRNA, IGF2BP2 siRNA, scrambled siRNA (NC), plasmid vector (Vector), or IGF2BP2-overexpressing construct (pcDNA4-IGF2BP2). (B, D) Typical results (B) and statistical analysis (D) of Transwell insert assay in HTR8/SVneo cells following transfection with MNSFβ siRNA together with or without IGF2BP2-overexpressing construct (pcDNA4-IGF2BP2). Scale bars indicate 100 μm. In each group, 10 views were randomly selected and the invaded cells were quantified. Data are presented as Mean ± SEM based on three independently repeated experiments, comparison between groups is carried out with Student's t-test. *, P < 0.05, **, P < 0.01.


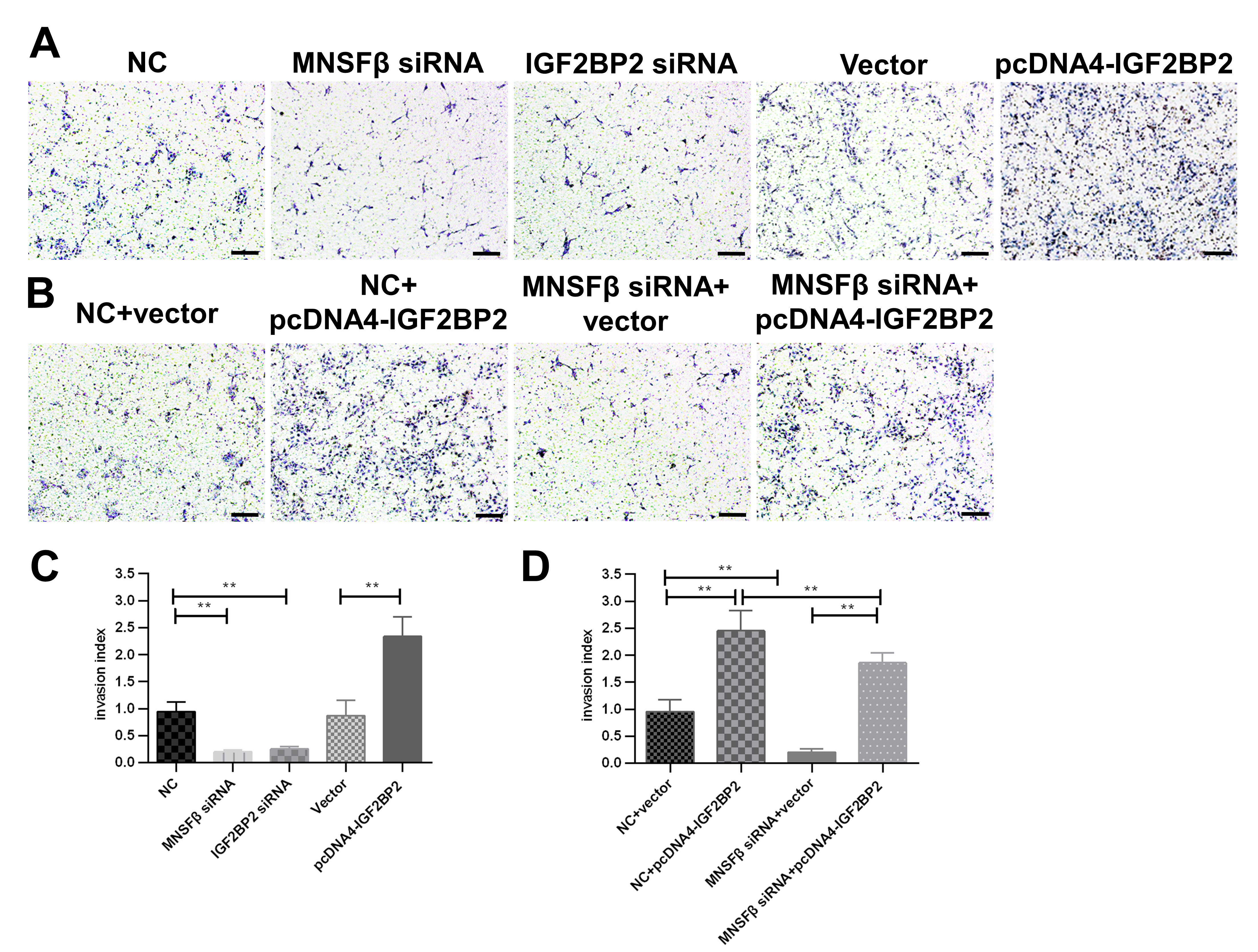


**Figure S3.** CCK-8 assay to measure cell viability in the same batch of JEG3 (A and B) and HTR8/SVneo (C and D) cells that were subjected to Transwell insert assay as shown in Figure 6 and Figure S2.


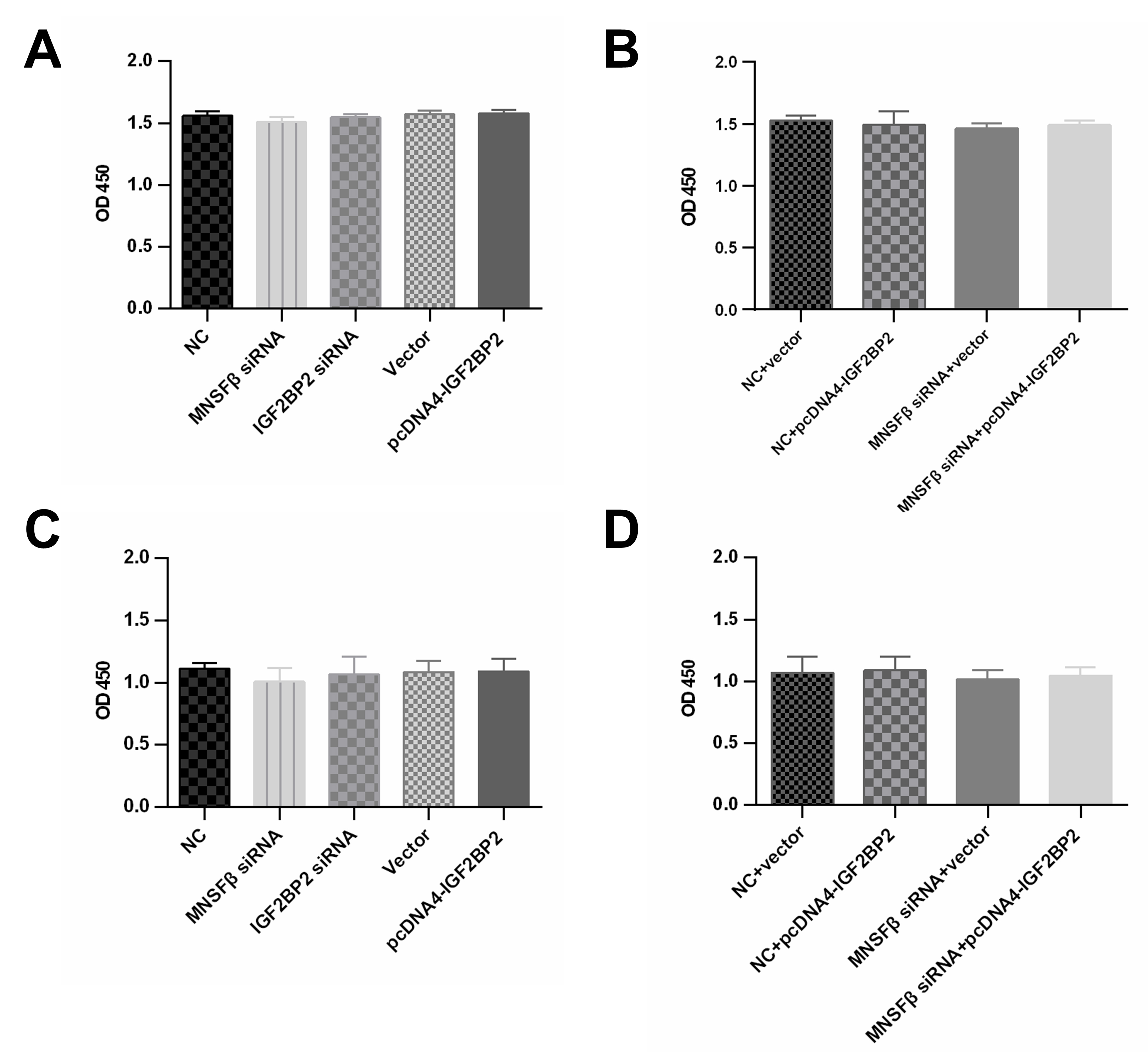


**Figure S4. Identification the ubiquitination of IGF2BP2 in cKO mice or severe PE placentas.** (A and B) 2 pairs of Control versus cKO mice (A) or PTL controls versus severe PE (B) placenta tissue lysates were immunoprecipitated with either an antibody against IGF2BP2 or an IgG control and then analyzed by immunoblotting with ubiquitin (Ub)-specific antibody and IGF2BP2 antibody.


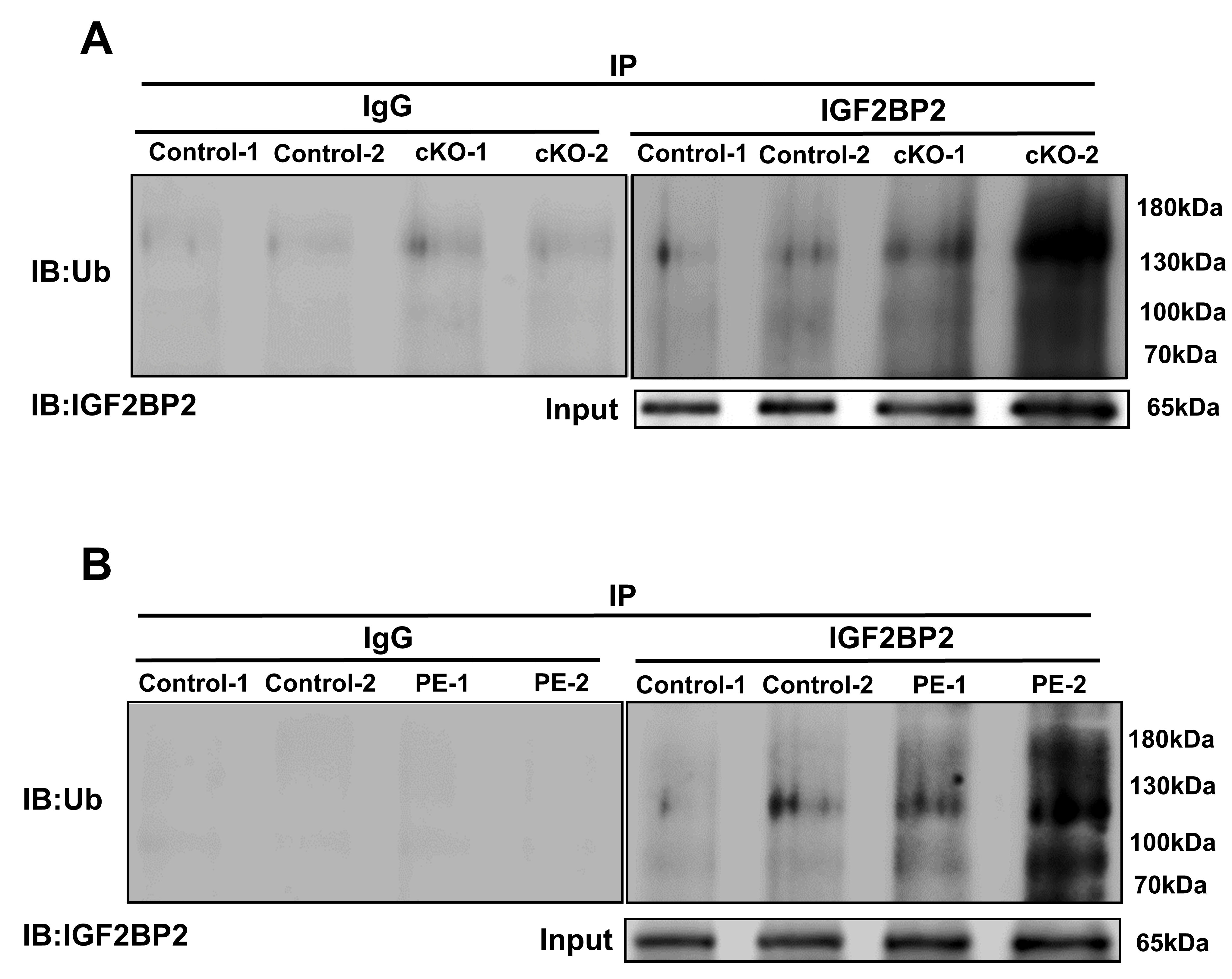

Supplement: Supplementary file 1 — Supplementary Material [file CPR-54-e13145-s001.docx]
